# Nanolipsome Modified with Inulin and Pea Protein Isolate Improve the Thermal Stability and Slow the Release of Anthocyanin at Simulated In Vitro Digestion and Hot Cocoa Beverage

**DOI:** 10.3390/foods14050731

**Published:** 2025-02-21

**Authors:** Aniya, Lianlian Zhang, Yan Li, Xiaoji Fu

**Affiliations:** 1 Institute of Agricultural Processing, Jiangxi Academy of Agricultural Sciences, Nanchang 330299, China; any2014@foxmail.com; 2 State Key Laboratory of Food Science and Resources, Nanchang University, Nanchang 330047, China; zll2422149552@163.com (L.Z.); yanli@ncu.edu.cn (Y.L.)

**Keywords:** anthocyanin nanoliposomes, double-layer modification, pea protein isolate, inulin, thermal stability, in vitro release

## Abstract

Anthocyanin (ACN) is a natural pigment with various biological activities, but their stability is compromised by external environmental factors, which limits their practical application in food processing. To enhance the stability of anthocyanin, double-layer-modified anthocyanin nanoliposomes (ACN-NLs) were prepared in this study using pea protein isolate (PPI) and inulin (IN) through layer-by-layer assembly in this study. The preparation conditions of unmodified, single-modified, and double-layer-modified nanoliposomes (ACN-NLs, PPI-ACN-NLs, and IN-PPI-ACN-NLs) were optimized via analysis of their average particle size, zeta potential, and encapsulation efficiency (EE). In addition, the structure of the nanoliposomes was characterized via transmission electron microscopy (TEM) and a Fourier transform infrared (FTIR) spectrometer. Furthermore, the thermal stability of nanoliposomes in hot cocoa and their release behavior during in vitro simulated digestion were evaluated. The results indicated that the optimal formulation for IN-PPI-ACN-NLs was 6% PPI and 2% IN. Under these conditions, the IN-PPI-ACN-NLs had a particle size of 270.2 ± 0.66 nm, a zeta potential of −15.76 ± 0.81 mV, and a high EE of 88.6 ± 0.71%. TEM analysis revealed that IN-PPI-ACN-NLs exhibited a spherical core–shell structure, while FTIR confirmed the interaction between ACNs and the encapsulating materials (PPI and IN). Compared with unmodified or monolayer-modified nanoliposomes, IN-PPI-ACN-NLs exhibited thermal stability in beverage systems and enhanced DPPH radical scavenging activity. During in vitro digestion, IN-PPI-ACN-NLs demonstrated a sustained-release effect and improved the digestive stability of ACN. These properties make it a promising functional additive for applications in the food and pharmaceutical industry.

## 1. Introduction

Red raspberry (*Rubus idaeus* L.) is a common berry that grows in the southern regions of China, and its fruit is rich in anthocyanin (ACN) [1]. ACNs are water-soluble polyphenolic compounds with various biological activities, including antioxidant, anti-inflammatory, anti-cancer, and anti-diabetes effects [2]. However, ACN has low lipid solubility, which makes it difficult for them to penetrate the phospholipid bilayer and enter cells, resulting in low bioavailability in the human body [3]. While ACNs can be directly absorbed by the small intestine, they struggle to withstand the harsh gastric environment [4]. In addition, ACNs are sensitive to high temperatures, pH changes, and light during food processing and storage, which can lead to a loss of biological activity [5].

In recent years, the use of nanoparticle technology has gained significant attention for improving the bioavailability of ACN. Nanoliposomes, as carriers of bioactive substances, enhance the solubility and stability of core materials, while offering targeted delivery and controlled release properties [6]. However, the nanoliposome membrane is susceptible to hydrolysis and damage from external factors, leading to reduced stability and poor absorption efficiency of anthocyanin [7]. Modification of nanoliposomes with macromolecules such as proteins and polysaccharides is considered a strategy to reduce the degradation of nanoliposomes in the environment and enhance the stability of nanoliposomes during storage [8]. This modification can also help regulate the penetration of phospholipid bilayers. Studies have shown that liposomes coated with whey protein isolate exhibit good storage stability and resistance to acidic environments [9]. Tai et al. [10] investigated the effects of chitosan modification on the stability and in vitro digestion of curcumin liposomes and found that increasing the molecular weight and concentration of chitosan helps delay the release of curcumin during in vitro digestion. However, monolayer-modified liposomes have a relatively loose structure, which limits their stability and sustained-release performance over time. To address this, layer-by-layer (LBL) self-assembly technology is commonly used for secondary coating to enhance its properties. LBL assembly involves the sequential deposition of polymers onto the liposome surface using intermolecular forces such as electrostatic attraction, covalent bonding, and hydrogen bonding, which forms a bilayer nanoscale coating [11]. This technique helps prevent direct contact between the phospholipids in nanoliposomes and the external environment, thereby reducing oxidative damage and hydrolysis. The double-layer modification increases the steric hindrance of liposomes and further prevents polymerization between liposome particles. Additionally, polymers can form electrostatic bridges between phospholipids and polymer molecules, reduce the permeability of the phospholipid bilayers, and enhance the stability of liposome systems [12]. Ghaleshahi et al. [13] applied layer-by-layer assembly using pectin and chitosan to modify perilla oil nanoliposomes. Transmission electron microscopy analysis revealed a typical core–shell structure, and the study found that these nanoliposomes could be digested by hydrolytic enzymes in the colon microbiota and fully absorbed in the colon.

Currently, the primary types of liposome coating materials include polysaccharides, animal proteins, and polymers such as polyethylene glycol. However, there are limited studies on the use of plant proteins in liposome coatings. Pea protein isolate (PPI), a valuable plant protein source, offers advantages such as low allergenicity and high nutritional value [14]. The isoelectric point of PPI typically ranges from 4 to 5 and gives it a positive charge below this range and a negative charge above it, thus demonstrating charge adjustability. This property allows PPI to bind to negatively charged liposome membranes and form a protective polyelectrolyte layer through electrostatic interactions [15]. In addition, PPI exhibits significant oxidation inhibition during liposome storage [16]. Inulin (IN) is a linear, anionic polysaccharide composed of fructose units linked by β-(2,1)-glycosidic bonds. It is found in plants such as chicory and Korean thistle and is known for its ability to enhance the texture and processing performance of food [17]. The human digestive system lacks enzymes capable of breaking down β-(2,1) linkages; hence, IN reaches the intestine intact, where it serves as a substrate for gut microbiota. Consequently, IN is widely used as a functional food ingredient [18]. Inulin can form a hydrogel, and the stability of its hydrogel structure can be improved by ultrasonic treatment, high-pressure homogenization, high-pressure hydrostatic pressure, and other means. Xue et al. [19] found that after ultrasonic treatment of 1734.9 w/cm^2^ intensity, the particle size distribution of inulin was more uniform, and it was more conducive to the generation of hydrogen bonds, thus enhancing the gel structure of long-chain inulin. Flowska et al. [20] reported that the stable gel structure of inulin was formed by high-pressure homogenization, which is mainly due to the hydrogen bonding and van der Waals interactions between dispersed (molecular polymer) particles during the process of high-pressure homogenization. Yamamoto et al. [21] found that high-pressure hydrostatic pressure could destroy the original polymer molecular structure of inulin, thus forming a more stable gel. In addition, PPI and IN can form biopolymers through a hydrogel network structure, which makes them effective carriers for delivering natural active substances [22]. Previous research has also shown that IN can improve the surface activity and emulsifying properties of β-lactoglobulin while enhancing protein stability [23].

Surface-modified liposomes have been recognized as a promising strategy for delivering bioactive substances. However, previous studies have not reported the preparation of anthocyanin-loaded nanoliposomes using PPI, IN, and layer-by-layer self-assembly modification technology. This study aims to develop anthocyanin-loaded nanoliposomes using PPI and IN through layer-by-layer modification to enhance the thermal stability of anthocyanins and slow their digestion in simulated stomachs and small intestines. In this study, anthocyanin nanoliposomes were prepared via the anti-solvent method, with an IN-PPI biopolymer as a mixed coating to produce double-modified nanoliposomes loaded with ACN. The nanoparticles were characterized using a particle size potential analyzer and through Fourier transform infrared (FTIR) spectrometry and transmission electron microscopy (TEM). Moreover, the thermal stability of ACN nanoliposomes in a hot instant cocoa beverage and their release behavior during simulated in vitro digestion were evaluated. This work provides a foundation for the rational design of novel nanoliposome delivery systems, expands the application of PPI and IN as biopolymer coating materials, and offers new insights into the use of ACN in food processing and medicine.

## 2. Materials and Methods

### 2.1. Materials

Red berry (*Rubus idaeus* L.) anthocyanins (ACN) powder (40%) was purchased from Ruimao Biotechnology Co., Ltd. (Nanjing, China). Soy lecithin (>75%), cholesterol (>90%), and ethanol (>90%) were obtained from Wokai Biotechnology Co., Ltd. (Beijing, China). Pea protein isolate, inulin (>80%), pepsin (activity: 3000–3500 NF), trypsin (>3000 unit/g), and bile salts were supplied by Yuanye Biotechnology Co., Ltd. (Shanghai, China). 6-Hydroxy-2,3,7,8-tetramethylchroman-2-carboxylic acid (Trolox, >90%), 2,2′-azino-bis(3-ethylbenzothiazoline-6-sulfonic acid) (ABTS, >90%), and 2,2-diphenyl-1-picrylhydrazyl (DPPH, >90%) were purchased from Aladdin Biochemical Technology Co., Ltd. (Shanghai, China). Dipotassium hydrogen phosphate (K_2_HPO_4_, >90%), glacial acetic acid (CH_3_COOH, >90%), concentrated hydrochloric acid (HCl, >90%), sodium hydroxide (NaOH, >90%), and other analytical-grade reagents were obtained from China National Pharmaceutical Co., Ltd. (Beijing, China).

### 2.2. Methods

#### 2.2.1. Preparation of ACN Nanoliposome (NL) Without Modification

Unmodified ACN nanoliposomes (ACN-NLs) were prepared via the anti-solvent method [24]. Firstly, 240 mg of phospholipids and 60 mg of cholesterol were dissolved in 15 mL of ethanol, and Tween-80 was thoroughly mixed to obtain the organic phase. A PBS solution (0.2 M, pH 3.5) containing 4 mg/mL ACN was prepared as the aqueous phase. The organic phase was rapidly injected into the aqueous phase and hydrated at 45 C for 30 min. The resulting mixture was then concentrated under reduced pressure using a rotary evaporator (RE-52A, Yarong Instruments Ltd., Shanghai, China) to completely remove the organic solvent. Finally, the cooled nanoliposome suspension was filtered through a 0.22 μm filter membrane (XH-LM, Huxi Instruments Ltd., Shanghai, China) to obtain unmodified ACN-NLs.

#### 2.2.2. Preparation of Solution of Pea Protein Isolate (PPI) and Inulin (IN)

A stock solution of PPI (10%, *w*/*v*) and inulin (10%, *w*/*v*) was prepared. Both solutions were stirred overnight and then sonicated for 15 min (40 kHz, 25 °C) using an ultrasonic water bath. After sonication, the solutions were stored at 4 °C until use.

#### 2.2.3. Preparation of ACN Nanoliposome (ACN-NL) with Modification by PPI and IN

The ACN nanoliposomes (ACN-NLs) modified with PPI and IN were prepared following the method of Liu et al. [25] with some modifications. To produce PPI-coated ACN-NLs (PPI-ACN-NLs), the different concentrations of PPI (0.5%, 1.0%, 2.0%, 4.0%, 6.0%, 8.0%, and 10.0%, *w*/*v*) were prepared by diluting the PPI stock solution (10.0% *w*/*v*) with acetate buffer solution (0.05 M, pH 3.5). ACN-NLs were then added dropwise to the PPI solution at a 1:1 (*v*/*v*) ratio. To determine the optimal IN concentration, different IN solutions (0.5%, 1.0%, 2.0%, 4.0%, 6.0%, 8.0%, and 10.0% *w*/*v*) were prepared by diluting the IN stock solution (1% *w*/*v*) with acetate buffer solution (0.05 M, pH 3.5). Afterward, PPI-ACN-NLs and IN solutions at different concentrations were mixed at a 1:1 (*v*/*v*) ratio with continuous stirring using a magnetic stirrer (IKA RCT basic, IKA Co., Ltd., Staufen, Germany) to form IN-coated PPI-ACN-NLs (IN-PPI-ACN-NLs). During this process, the particle size, polydispersity index (PDI), and zeta potential were measured using a zetasizer (Nano-ZS90, Malvern Instruments Ltd., Worcestershire, UK) to obtain the optimum concentrations of PPI and IN for stable IN-PPI-ACN-NLs.

#### 2.2.4. Characterization of Different Nanoliposomes

##### Particle Size, Zeta Potential, and Polydispersity Index (PDI)

The particle size, zeta potential, and dispersion coefficient (PDI) of the samples were measured via the dynamic light scattering (DLS) method with a zetasizer (Nano-ZS90, Malvern Instruments Ltd., Worcestershire, UK). The dispersion medium was water, the test temperature was set to 25 °C, the scattering angle was set at 90°, and the equilibrium time was 120 s. Before testing, the ACN-NL, PPI-ACN-NL, and IN-PPI-ACN-NL samples were diluted 100-fold, 100-fold, and 50-fold, respectively [26]. Water was used as the dispersion medium, the test temperature was set to 25 °C, the scattering angle was set to 90°, and the equilibrium time was 120 s. Before testing, the ACN-NL, PPI-ACN-NL, and IN-PPI-ACN-NL samples were diluted 100-fold, 100-fold, and 50-fold, respectively [26].

##### Microscopic Morphology Under Transmission Electron Microscopy (TEM)

The ACN-NL, PPI-ACN-NL, and IN-PPI-ACN-NL samples were diluted 10-fold and placed on a copper mesh. The samples were then stained with 1% phosphotungstic acid for 5 min and dried at room temperature. The morphology of the liposomes was observed using a TEM (Talos L120c, Thermo Fisher Scientific, Waltham, MA, USA) at 120 KV [27].

##### Determination of Encapsulation Efficiency (EE%) and ACN Release Rate

A 4.0 mL sample of ACN-NLs was centrifuged using a centrifuge (Avanti J-E, Beckman Coulter Inc., Brea, CA, USA) at a speed of 15,000 r/min for 30 min. An equal volume of acidic ethanol solution was added to break the emulsion, and the supernatant was collected to determine the ACN content. The ACN content was measured via the pH differential method [28]. Specifically, 4.5 mL of pH 1.0 potassium chloride–hydrochloric acid buffer solution (0.4 M) was each added to 0.5 mL of the supernatant. The absorbance values at the maximum absorption wavelengths of 520 nm and 700 nm were measured using a microplate reader (Spark 20 M, Tecan Austria GmbH, Grodig, Austria).

The encapsulation efficiency (EE%) of ACN was evaluated by measuring the total ACN content (C_1_) before encapsulation and the free ACN content (C_2_) in nanoliposomes after encapsulation, following the method of Ge et al. [4]. The encapsulation efficiency was calculated using the following equation (Equation (1)):

(1) $$EE\%=(C_{1}-C_{2})\times 100$$

C_1_ (mg/mL)—the total ACN content before encapsulation;

C_2_ (mg/mL)—the free ACN content in nanoliposomes after encapsulation.

The release rate of ACN was determined by measuring the initial ACN content in the sample (C_0_) and the ACN content at a specific time (C_t_), following the method of [4]. The release rate was calculated using the following equation (Equation (2)):

(2) $$ACN\;release(\%)=(1-C_{t}/C_{0})\times 100$$

C_0_ (mg/mL)—the content of ACN in the sample at the initial time (C_0_);

C_t_ (mg/mL)—the content of ACN in the sample at a certain time (C_t_).

##### Characterization of Fourier Transform Infrared Spectroscopy (FTIR)

An FTIR spectrometer (Nicolet iS10, Thermo Scientific, New York, NY, USA) was used to confirm the binding of biopolymers (PPI and/or IN) to nanoliposomes (NLs). According to the method of Katouzian et al. [29], samples of ACN-NLs, PPI-ACN-NLs, and IN-PPI-ACN-NLs were vacuum freeze-dried at −80 °C overnight. by a vacuum freeze-dryer (GAMMA 1–16 LSC; Osterode, Germany). Each sample was mixed with anhydrous KBr and the mixtures were compressed into thin slices. The structural characteristics of the NLs and their coating materials were then analyzed via FTIR over a wavelength range of 400 cm^−1^ to 4000 cm^−1^ with a resolution of 4 cm^−1^ in an attenuated total reflection (ATR) mode and the signal was transformed to transmittance.

##### Determination of DPPH Radical Scavenging Activity (DPPH-RSA)

The determination of DPPH radical scavenging activity (DPPH-RSA) was based on the method of Gorjanovic et al. [30], with appropriate modifications. A 300 μL sample solution or Trolox standard solution (25–200 ppm) was added to 1.9 mL of 0.093 mmol/L DPPH solution. After thorough mixing, the solution was kept in the dark for 60 min. The absorbance at 517 nm was then measured using a microplate reader (Spark 20 M, Tecan Austria GmbH, Grodig, Austria). DPPH-RSA was expressed in terms of Trolox equivalents per gram of sample (mg TE eq/g).

#### 2.2.5. Addition of ACN Nanoliposomes (ACN-NLs) to Instant Hot Cocoa Beverage

Three types of ACN-NLs and an ACN solution (used as a control) were added to an instant hot cocoa beverage to evaluate the thermal stability of the mixed cocoa beverage system, as described in a previous study [31]. Instant cocoa powder (2 g) was mixed with 200 mL of the following solutions: distilled water (Sample 1, cocoa), ACN solution (4 mg/mL of ACN, Sample 2, cocoa/ACN), ACN-NLs (4 mg/mL of ACN, Sample 3, cocoa/ACN-NLs), PPI-ACN-NLs (4 mg/mL of ACN, Sample 4, cocoa/PPI-ACN-NLs) or IN-PPI-ACN-NLs (4 mg/mL of ACN, Sample 5, cocoa/IN-PPI-ACN-NLs). Each mixture was dispersed at 10,000 rpm for 4 min using a high-speed blender disperser (Ultra Turrax T-10, IKA Co., Ltd., Staufen, Germany) to obtain a homogeneous cocoa beverage sample. The prepared beverage samples were then heated at 50 °C, 70 °C, or 90 °C for 3 h before analyzing ACN release and DPPH-RSA.

#### 2.2.6. Release of ACN During In Vitro Simulated Digestion

Simulated gastric fluid (SGF) and simulated intestinal fluid (SIF) were prepared following the method of Brodkorb et al. [32], with slight modifications. SGF was prepared by dissolving 0.2 g of sodium chloride, 0.36 g of gastric protease, and 0.7 mL of hydrochloric acid in water to a total volume of 100 mL to adjust the pH to approximately 2.5. SIF was prepared by dissolving 1 g of trypsin and 5 g of bile salt in 100 mL of 5 mmol/L potassium dihydrogen phosphate buffer solution to adjust the pH to 7. The release behavior of ACN-NLs during in vitro simulated digestion was assessed via a modified version of the method described by Liu et al. [25]. A total of 30 mL of ACN, ACN-NLs, PPI-ACN-NLs, or IN-PPI-ACN-NLs was mixed with an equal volume of SGF or SIF. Finally, 2 mL of SGF or SIF mixture was taken at 0, 30 min, 60 min, 90 min, 120 min, 150 min, and 180 min to measure the release rate of ACN. In addition, kinetic fitting analysis was performed to evaluate the release behavior of the samples during in vitro digestion. The zero-order model, first-order model, Higuchi model, and Ritger–Peppas model were used to investigate the release behavior of anthocyanins from ACNs, ACN-NLs, PPI-ACN-NLs, and IN-PPI-ACN-NLs during in vitro digestion [33]. These four models were fitted using Origin 2019 software, and their formulations are as follows:

$$\mathrm{Zero}-\mathrm{order}\;\mathrm{model}\text{:}\;\frac{M_{t}}{M_{0}}=kt$$

$$\mathrm{First}-\mathrm{order}\;\mathrm{model}\text{:}\;\ln\left(1-\frac{M_{t}}{M_{0}}\right)=-kt$$

$$\mathrm{Higuchi}\;\mathrm{model}\text{:}\;\frac{M_{t}}{M_{0}}=kt^{0.5}$$

$$\mathrm{Ritger}\text{–}\mathrm{Peppas}\;\mathrm{model}\text{:}\;\frac{M_{t}}{M_{0}}=kt^{n}$$

where M_t_ and M_0_ represent the actual and theoretical release amounts of ACN at time t, respectively. k is the rate constant of the equation, and n is the release exponent, which characterizes the release mechanism. Generally, when n < 0.5, the release follows Fickian diffusion, whereas when 0.5 < n < 1, it follows non-Fickian diffusion.

#### 2.2.7. Statistical Analysis

All experiments were conducted in triplicate, and the results were expressed as “mean ± standard deviation”. Data analysis was performed using SPSS software (IBM SPSS Statistics 26) with Duncan’s test and analysis of variance (ANOVA) for statistical evaluation. Graphs were generated using Origin 9.0 software. A significance level of *p* < 0.05 was considered statistically significant.

## 3. Results and Discussion

### 3.1. Optimization of Preparation Conditions for IN-PPI-ACN-NL

In this study, the particle size, PDI, zeta potential, and EE% of nanoliposomes bound with different concentrations of PPI/In were evaluated via the method proposed by Chun et al. [34], while optimizing the PPI/In concentrations. The volume ratio of the fixed PPI solution to unmodified ACN-NLs was 1:1. Table 1 presented the average particle size, PDI, zeta potential, and EE% of PPI-ACN-NLs prepared at different PPI concentrations. According to Table 1, the particle size of unmodified ACN-NLs (0% PPI-ACN-NLs) was 174.7 ± 2.14 nm, with a zeta potential of −16.6 ± 0.21 mV and a PDI of 0.18 ± 0.003, while the EE% was only 61.7 ± 0.83%. As the PPI concentration increased from 0% to 10.0%, the particle size of PPI-ACN-NLs increased to 295.6 ± 1.71 nm, and the PDI rose to 0.40 ± 0.018. This increase might be due to the higher PPI concentration, which led to the thickening of the PPI adhesion layer on the liposome surface. Similarly, Nguyen et al. [35] modified berberine liposomes with chitosan, which resulted in a significant increase in particle size and indicated that chitosan formed a coating on the surface of the liposomes.

After single-layer modification with PPI, the zeta potential of ACN-NLs shifted from negative to positive and reached 23.70 ± 0.31 mV. This shift indicated that ACN-NLs carried a negative charge, while PPI molecules in solution carried a positive charge. Consequently, PPI could adsorb onto the surface of liposomes through electrostatic interactions, which made them positively charged [36].

As the concentration of PPI increased from 0% to 6%, the EE% of anthocyanins rose from 61.7 ± 0.83% to 82.9 ± 0.51%. This increase might be because, within an appropriate PPI concentration range, PPI formed a protective barrier on the surface of liposomes, strengthened molecular interactions, and prevented ACN leakage from the liposome sublayer [37]. However, when the PPI concentration exceeded 6%, the EE% began to decline. Research has shown that excessively high concentrations of proteins or polysaccharides can lead to the spontaneous aggregation and coagulation of hydrophobic groups on their molecular chains and hinder the adsorption of macromolecules on the surface of nanoliposomes, which reduces their stability [38]. According to a comprehensive analysis, the optimal PPI concentration was determined to be 6%. At this concentration, the EE% reached 82.9 ± 0.51%, the particle size was 266.2 ± 2.70 nm, the PDI was 0.32 ± 0.020, and the zeta potential was 21.82 ± 0.77 mV, which indicated that the nanoliposome system was in a relatively stable state.

According to the optimized preparation of 6.0% PPI-ACN-NLs, different concentrations of IN solutions (0.5%, 1.0%, 2.0%, 4.0%, 6.0%, 8.0%, and 10.0%) were used for the second-layer modification of liposomes, which resulted in seven types of IN-PPI-ACN-NLs. The volume ratio of the IN solution to PPI-modified PPI-ACN-NLs was fixed at 1:1. The average particle size, PDI, ζ-potential, and encapsulation efficiency of bilayer-modified IN-6% PPI-ACN-NLs at different IN concentrations are shown in Table 2. The results in Table 2 indicate that the particle size of IN-PPI-ACN-NLs is positively correlated with the concentration of IN. As the IN concentration increased from 0.5% to 10%, the particle size increased from 259.4 ± 1.82 nm to 327.2 ± 3.51 nm.

The PDI value initially decreased and then increased as the IN concentration increased, which ranged from 0.36 ± 0.016 to 0.43 ± 0.030. This trend suggested that an excessive IN concentration negatively affected the stability of the nanoliposome system. One possible explanation was that at higher IN concentrations, IN binds to the PPI on the liposome surface and displaces the PPI previously attached to the exposed liposomes. This displacement created instability during the separation process, which led to a disordered system. At this stage, while PPI did not completely detach from the liposomes, it was bound in excessive amounts and resulted in larger particle sizes. This observation aligned with the findings of Villiers et al. [39] and others.

Without IN modification, the zeta potential of PPI-ACN-NLs was 21.82 ± 0.77 mV. However, after the addition of IN, the zeta potential of IN-PPI-ACN-NLs decreased to −18.25 ± 0.71 mV. This change might be attributed to the formation of a new protective layer on the surface of PPI-ACN-NLs by negatively charged IN through secondary electrostatic interactions. Similarly, Barbosa et al. [40] reported that sodium alginate, an anionic polysaccharide, caused the zeta potential of chitosan-modified nanoliposomes to shift from positive to negative. The observed change in zeta potential further confirmed the successful encapsulation of PPI and IN on the surface of ACN-NLs through layer-by-layer modification.

As the concentration of IN increased, its EE% initially rose and then declined. At an IN concentration of 2%, IN-PPI-ACN-NLs achieved the highest EE% of 88.6 ± 0.71%. However, the overall variation in encapsulation efficiency was not significant, which suggested that IN concentration had minimal impact on the EE% of liposomes. This observation indicated that double modification might not alter the internal structure of liposomes; instead, IN formed an additional physical barrier on the surface of PPI-ACN-NLs, which enhanced the structural stability of the liposomes [41]. Considering both EE% and system stability, 2% IN concentration was selected for the secondary modification of PPI-ACN-NLs, yielding IN-PPI-ACN-NLs with an EE% of 88.6 ± 0.71%.

### 3.2. Structural Characterization of PPI-IN-Modified Anthocyanin Nanoliposomes

#### 3.2.1. Observation of Micro-Structure

**Figure 1:**
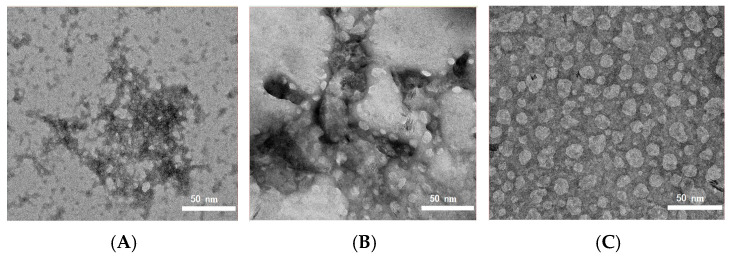
TEM images of (**A**) anthocyanin nanoliposomes (ACN-NLs); (**B**) pea protein isolate-coated ACN-NLs (PPI-ACN-NLs); and (**C**) inulin-coated pea protein isolate–ACN-NLs (IN-PPI-ACN-NLs).

TEM analysis was conducted to examine the morphology of the nanoliposomes. The TEM images of ACN-NLs, PPI-ACN-NLs, and IN-PPI-ACN-NLs are shown in Figure 1. The vesicles of ACN-NLs (without PPI modification) and PPI-ACN-NLs (with monolayer modification) appeared as smaller, spherical structures with a more concentrated distribution. In contrast, IN-PPI-ACN-NLs, which underwent dual modification, exhibited a significant increase in particle size and a distinct core–shell structure. The inner layer appeared bright white with high saturation, while the outer layer was covered with a coating. This coating is speculated to be a vesicular structure formed by the interaction between phospholipids and PPI, as well as between PPI and IN [42]. Similar TEM imaging results have been reported in previous studies on liposomes prepared using soy lecithin and cinnamon oil. Researchers suggested that protein–polysaccharide complexes used as liposome coatings could create physical barriers on the surface of nanoparticles, enhance colloidal stability, and improve the controlled release of encapsulated materials [43].

#### 3.2.2. Analysis of FTIR

**Figure 2:**
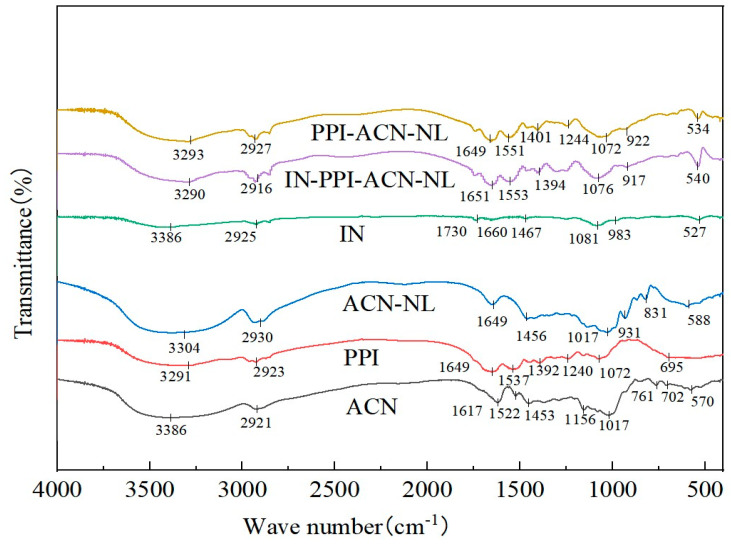
FTIR spectra of anthocyanins (ACN), anthocyanin nanoliposomes (ACN-NLs), pea protein isolate (PPI), inulin (IN), pea protein isolate-coated ACN-NLs (PPI-ACN-NLs), and inulin-coated pea protein–ACN-NLs (IN-PPI-ACN-NLs).

FTIR spectrometry can be used to determine the chemical structures of ACN-NLs, PPI-ACN-NLs, and IN-PPI-ACN-NLs, which enables the identification of modifications in chemical bonds and confirms the successful coating of the nanoliposome’s outer layer with PPI and IN. The FTIR spectra of ACN-NLs, PPI-ACN-NLs, IN-PPI-ACN-NLs, PPI, and IN are shown in Figure 2. As seen in Figure 2, the C-N vibration peak of ACNs at 1016 cm^−1^ shifted to 931 cm^−1^ in ACN-NLs, while the C=C peak at 1552 cm^−1^ did not exhibit a distinct characteristic absorption peak in ACN-NLs, which indicated the successful encapsulation of ACNs within the liposome core [44]. The N-H vibration peak of PPI at 1537 cm^−1^ was a characteristic peak of its amino structure. In PPI-ACN-NLs, this peak shifted to 1551 cm^−1^, which indicated electrostatic crosslinking between PPI and the liposomes. The broad absorption band of inulin at 3600–3100 cm^−1^ corresponded to the O-H stretching vibration and hydrogen bonding of hydroxyl groups. Compared with IN, the absorption peak of IN-PPI-ACN-NLs around 3290 cm^−1^ was enhanced, likely owing to the introduction of polysaccharides causing O-H stretching or N-H deformation vibrations [45]. In nanoliposomes, the symmetric and asymmetric vibrational modes of CH_2_ in the fatty acid hydrocarbon chains, which form the hydrophobic tail of phospholipids, were located at 2927 cm^−1^ and 2916 cm^−1^, respectively. However, these peak positions remained unchanged in PPI-ACN-NLs and IN-PPI-ACN-NLs, which indicated that PPI and IN interacted with phosphate groups on the liposome surface through electrostatic crosslinking and hydrogen bonding. This surface modification did not alter the internal structure of the liposomes [46].

### 3.3. Thermal Stability of Nanoliposomes in Hot Cocoa Beverage System

**Figure 3:**
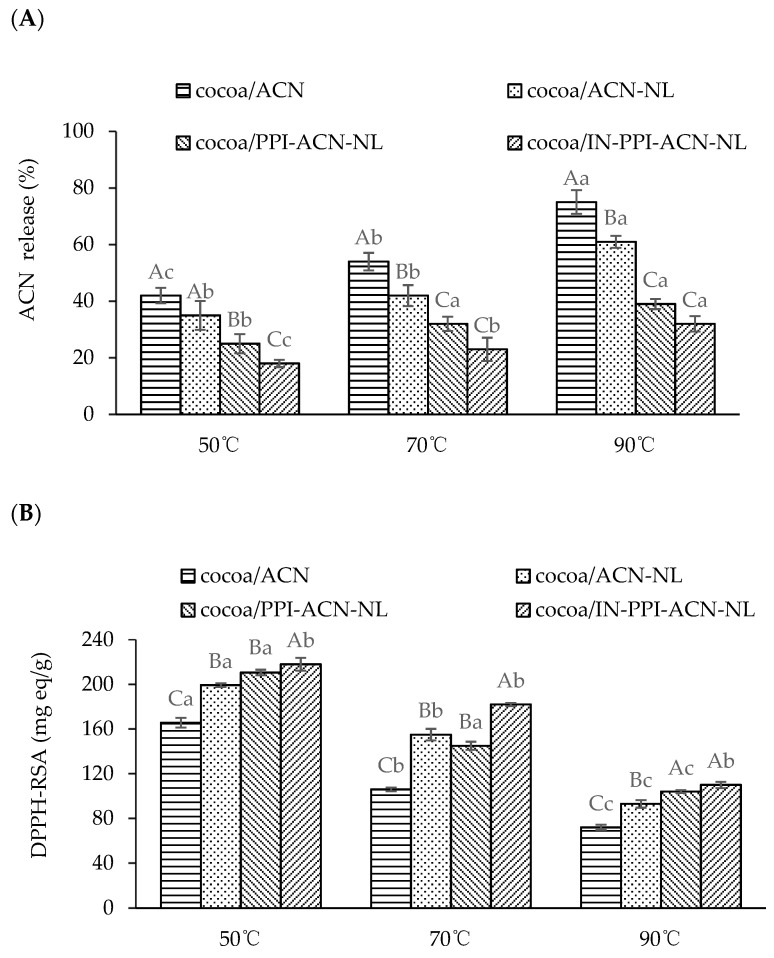
(**A**) Release of the ACN in the hot instant cocoa beverages prepared with four anthocyanin formulas (cocoa/ACN, cocoa/ACN-NLs, cocoa/PPI-ACN-NLs, cocoa/IN-PPI-ACN-NLs); (**B**) DPPH free radical scavenging activity of the hot instant coffee beverages prepared with four anthocyanin formulas (cocoa/ACN, cocoa/ACN-NLs, cocoa/PPI-ACN-NLs, cocoa/IN-PPI-ACN-NLs). Different capital letters indicate significant differences between different anthocyanin formulas (*p* < 0.05). Different lowercase letters indicate significant differences under different temperatures (*p* < 0.05).

In food production, high-temperature heating is an effective method for sterilization and further processing. However, previous studies have shown that ACN are highly susceptible to degradation at high temperatures, leading to the loss of various biological activities, including antioxidant properties. This study measured the antioxidant activity and degradation of ACN in a cocoa beverage system after the addition of ACN and ACN-NLs to evaluate the thermal stability of the system. The degradation rates of ACN in cocoa/ACN, cocoa/ACN-NLs, cocoa/PPI-ACN-NLs, and cocoa/IN-PPI-ACN-NLs after heating at 50 °C, 70 °C, and 90 °C for 3 h are shown in Figure 3A. After heating at 50 °C for 3 h, the degradation rates of ACN in cocoa/ACN and cocoa/ACN-NLs were 42.06 ± 2.71% and 35.12 ± 4.10%, respectively, while those in cocoa/PPI-ACN-NLs and cocoa/IN-PPI-ACN-NLs were only 25.50 ± 3.04% and 18.62 ± 1.31%, respectively. At 70 °C, the degradation rate of ACN in cocoa/ACNs increased to 54.28 ± 3.13%, whereas the degradation rates in cocoa/PPI-ACN-NLs and cocoa/IN-PPI-ACN-NLs remained lower at 34.06 ± 2.51% and 23.55 ± 4.10%, respectively. After heating at 90 °C, the degradation rate of ACN in cocoa/ACNs reached 75.03 ± 4.21%, whereas in cocoa/IN-PPI-ACN-NLs, it was only 31.22 ± 2.70%. Similarly, Wang et al. [47] reported that ACN-loaded nanocomplexes derived from chitosan and whey protein slowed down the release of anthocyanins in coffee. As the source of bioactive peptides and polysaccharides, PPI and IN may resist the influence of high temperatures according to the research of Soares et al. [48] and Tavares [49]. In addition, the thermal stability of ACN in nanoliposomes is also enhanced by hydrophobic interactions between the hydrophobic groups of the PPI and ACN [50].

The DPPH radical scavenging activity (DPPH-RSA) of hot instant coffee beverages prepared with four ACN formulations (cocoa/ACN, cocoa/ACN-NLs, cocoa/PPI-ACN-NLs, and cocoa/IN-PPI-ACN-NLs) is shown in Figure 3B. After heating at 50 °C for 3 h, the DPPH-RSA of cocoa/ACN was 165.72 ± 4.13 mg TE eq/g. The DPPH-RSA of cocoa/PPI-ACN-NLs and cocoa/IN-PPI-ACN-NLs was approximately 35 mg TE eq/g and 43 mg TE eq/g higher than that of cocoa/ACN, respectively. After heating at 90 °C for 3 h, the DPPH-RSA of cocoa/ACN decreased to 72.25 ± 2.33 mg TE eq/g, while the DPPH-RSA of cocoa/PPI-ACN-NLs and cocoa/IN-PPI-ACN-NLs remained at approximately 42 mg TE eq/g and 51 mg TE eq/g higher than that of cocoa/ACN at the same temperature. Previous studies have shown that the ACN nanocomposites composed of pea protein and inulin exhibited good dispersibility and stability in low-pH and heat-treatment systems, which was attributed to the electrostatic interactions between polysaccharides and proteins [22]. Compared with cocoa/ACN, the improved antioxidant performance of hot cocoa beverages was attributed to the enhanced stability of ACN in cocoa/ACN-NLs, cocoa/PPI-ACN-NLs, and cocoa/IN-PPI-ACN-NLs, which was consistent with the findings of Hu et al. [51]. Furthermore, the presence of PPI and IN in hot cocoa beverages containing PPI-ACN-NLs and IN-PPI-ACN-NLs might promote the Maillard reaction, and some Maillard reaction products could contribute to antioxidant activity [52]

### 3.4. Release Behavior of ACN During In Vitro Simulated Digestion

**Figure 4:**
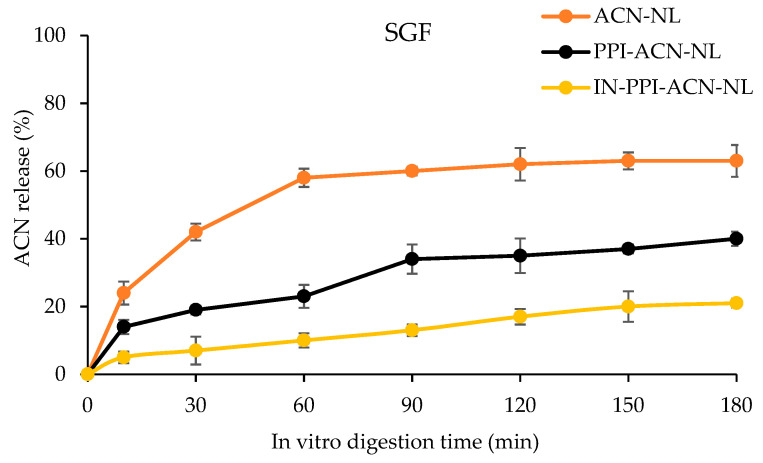
Anthocyanin (ACN) released from anthocyanin nanoliposomes (ACN-NLs), pea protein isolate-coated ACN-NLs (PPI-ACN-NLs), and inulin-coated pea protein isolate–ACN-NLs (IN-PPI-ACN-NLs) in simulated gastric digestion.

Previous studies have shown that nanoliposomes and ACN is easily degraded by strong acid or alkaline digestive fluids in the gastrointestinal tract, leading to the leakage of encapsulated compounds and reducing their bioavailability [53]. Therefore, this study conducted in vitro simulated digestion experiments on nanoliposomes before and after modification with IN and PPI to evaluate the release behavior of ACN during in vitro digestion. The release rates of ACN from ACN-NLs, PPI-ACN-NLs, and IN-PPI-ACN-NLs during simulated gastric digestion are shown in Figure 4. As illustrated in the figure, ACN release from the three nanoliposome formulations initially increased and then decreased over time. After 90 min of simulated gastric digestion, the release rate peaked for all three formulations. The release rate of ACN-NLs reached 59.40%, whereas PPI-ACN-NLs and IN-PPI-ACN-NLs exhibited significantly lower release rates of 34.06% and 13.25%, respectively. During the simulated gastric digestion stage, the release rate of ACN in ACN-NLs was significantly higher than that in PPI-ACN-NLs and IN-PPI-ACN-NLs. This observation might be due to inulin’s ability to reduce gastric protease activity [54]. Moreover, as reported by Yi et al. [55], proteins in gastric acid could slow down drug release. Consequently, IN-PPI-ACN-NLs demonstrated higher stability in gastric juice, which might facilitate the transport of more encapsulated ACNs to the small intestine.

**Figure 5:**
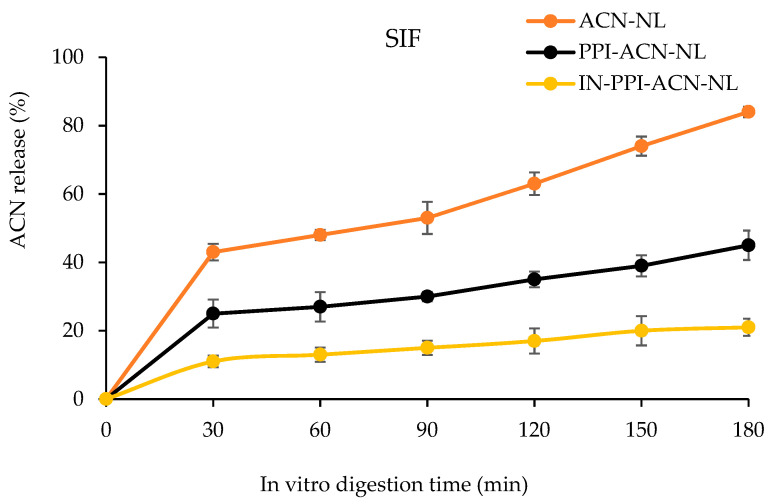
Anthocyanin (ACN) released from anthocyanin nanoliposomes (ACN-NLs), pea protein isolate-coated ACN-NLs (PPI-ACN-NLs), and inulin-coated pea protein isolate–ACN-NLs (IN-PPI-ACN-NLs) in simulated intestinal digestion.

The release rates of ACN from ACN-NLs, PPI-ACN-NLs, and IN-PPI-ACN-NLs during simulated intestinal digestion are shown in Figure 5. As depicted in Figure 5, the release rate of ACN in ACN-NLs exhibited a rapid increase over time. In contrast, ACN in PPI-ACN-NLs and IN-PPI-ACN-NLs followed a pattern of rapid initial release, followed by a slower release phase. During simulated intestinal digestion, the release rate of ACN in ACN-NLs reached a maximum of 84.10%, whereas the release rates in PPI-ACN-NLs and IN-PPI-ACN-NLs were only 45.62% and 21.78%, respectively. During the entire simulated intestinal digestion process, unmodified and monolayer-modified nanoliposomes released more ACN than bilayer-modified nanoliposomes. This observation might be due to the hydrolysis of phospholipids in the outer layer of liposomes by trypsin in intestinal fluid, as well as the solubilization effect of bile salts, which led to the rupture of liposomes and the subsequent release of a large amount of ACN. The PPI and IN encapsulated on the surface of liposomes could prevent pancreatic enzymes and bile salts from contacting the phospholipids on the outer layer, thereby maintaining the integrity of the liposomes [56]. Sahay et al. [57] found that nanoparticles could be directly absorbed by epithelial cells through active or passive transport mechanisms. Therefore, bilayer-modified nanoliposomes could significantly enhance the bioavailability of ACN.

The fitting results of ACN-NLs, PPI-ACN-NLs, and IN-PPI-ACN-NLs in different slow-release kinetic models during simulated digestion are shown in Table 3 and Table 4. The correlation coefficient (R^2^) indicated the degree of fit between the release behavior of ACN from nanoliposomes and the respective models. During the simulated gastric digestion stage, the release of ACN from ACN-NLs followed a first-order kinetic model, likely owing to the higher initial release rate of ACN. The release of ACN from PPI-ACN-NLs and IN-PPI-ACN-NLs best fits the Higuchi model, which describes a release rate proportional to time and is characteristic of a typical slow-release system. The gastric digestive environment, with its strong acids and digestive enzymes, tends to degrade the embedding system. However, compared with ACN-NLs, IN-PPI-ACN-NLs exhibited a more efficient ACN release, while PPI-ACN-NLs released less, which suggested that bilayer modification provided a sustained-release effect. This finding aligned with the results reported by Fang et al. [58]. During the small intestine digestion stage, the release of ACN from ACN-NLs followed the Ritger–Peppas model, with n < 0.43, which indicated Fickian diffusion, a steady-state diffusion process. In contrast, the release from PPI-ACN-NLs and IN-PPI-ACN-NLs followed the Higuchi model. Despite the complex intestinal environment, the single-layer and bilayer modifications in PPI-ACN-NLs and IN-PPI-ACN-NLs provided sustained release, which allowed the carrier material to adapt to the gastric environment while reducing the influence of various ions in the intestinal fluid. This finding is consistent with the results of Hasan et al. [59].

## 4. Conclusions

In this study, nanoliposomes modified with PPI and IN were successfully synthesized. The results showed that after double-layer modification with PPI and IN, the particle size of the nanoliposomes increased from 174.7 ± 2.14 nm to 270.2 ± 0.66 nm, the zeta potential changed from −16.6 ± 0.21 mV to −15.76 ± 0.81 mV, and the ACN encapsulation efficiency increased from 61.7 ± 0.83% to 88.6 ± 0.71%. These findings indicated that PPI and IN were successfully attached to the nanoliposome surface, and their interaction modified the original nanoliposome structure. The TEM results indicated that the IN-PPI-ACN-NLs obtained after double-layer modification exhibited a spherical core–shell structure. Changes in the absorption peak positions and intensities of various functional groups in Fourier transform infrared spectroscopy further confirmed the successful modification of PPI and IN on the nanoliposome surface. The thermal stability in hot cocoa and the in vitro release results from simulated gastrointestinal digestion demonstrated that PPI-IN bilayer modification significantly enhanced the thermal stability and antioxidant activity of ACN-NLs in hot beverage systems. In addition, this modification led to a significant reduction in the release rate of anthocyanins in the gastrointestinal tract. Therefore, in complex food processing systems, the use of PPI-IN bilayer-modified nanoliposomes could prevent the rapid release of ACN, promote their gastrointestinal absorption, and enhance the nutritional value of food. In conclusion, the combination of PPI and IN on the nanoliposome surface is a promising approach with significant application potential in the food, health products, and pharmaceutical industries. For future research, it is recommended to develop PPI-IN-modified nanoliposomes for food enrichment and assess their feasibility through sensory evaluation. The findings of this study provide valuable reference data for the application of PPI-IN bilayer-modified ACN nanoliposomes in the food industry and lay the foundation for their use in various food systems.

## Figures and Tables

**Table 1:** Mean particle size, PDI, ζ-potential, and EE% of PPI-ACN-NLs at different PPI concentrations. Different lowercase letters indicate significant differences (*p* < 0.05) among PPI-ACN-NL samples with varying PPI concentrations.

Nanoliposome	Mean Particle Size (nm)	PDI	ζ-Potential (mV)	EE (%)
0% PPI-ACN-NLs	174.7 ± 2.14 ^e^	0.18 ± 0.003 ^e^	−16.6 ± 0.21 ^f^	61.7 ± 0.83 ^d^
0.5% PPI-ACN-NLs	183.5 ± 3.02 ^d^	0.22 ± 0.008 ^d^	5.17 ± 1.08 ^e^	68.5 ± 0.27 ^c^
1.0% PPI-ACN-NLs	216.9 ± 1.75 ^c^	0.25 ± 0.017 ^d^	17.33 ± 0.45 ^d^	74.6 ± 0.44 ^b^
2.0% PPI-ACN-NLs	249.0 ± 5.20 ^c^	0.27 ± 0.005 ^c^	20.55 ± 0.92 ^b^	78.2 ± 1.21 ^b^
4.0% PPI-ACN-NLs	258.4 ± 0.13 ^b^	0.34 ± 0.016 ^a^	23.70 ± 0.31 ^a^	81.7 ± 0.97 ^a^
6.0% PPI-ACN-NLs	266.2 ± 2.70 ^b^	0.32 ± 0.020 ^b^	21.82 ± 0.77 ^a^	82.9 ± 0.51 ^a^
8.0% PPI-ACN-NLs	280.3 ± 3.44 ^a^	0.38 ± 0.035 ^a^	16.40 ± 1.13 ^d^	81.0 ± 0.37 ^a^
10.0% PPI-ACN-NLs	295.6 ± 1.71 ^a^	0.41 ± 0.018 ^a^	19.67 ± 0.71 ^c^	82.1 ± 0.33 ^a^

**Table 2:** Mean particle size, polydispersity index (PDI), ζ-potential, and encapsulation efficiency (EE) of different IN-PPI-ACN-NLs at different IN concentrations. The different lowercase letters indicated a significant difference (*p* < 0.05) between the IN-PPI-ACN-NLs with different concentrations of IN added.

Nanoliposome	Mean Particle Size (nm)	PDI	ζ-Potential (mV)	EE (%)
0.5% IN-6.0% PPI-ACN-NLs	259.4 ± 1.82 ^f^	0.36 ± 0.016 ^b^	−3.51 ± 0.55 ^a^	84.7 ± 0.33 ^b^
1.0% IN-6.0% PPI-ACN-NLs	268.5 ± 1.13 ^d^	0.34 ± 0.007 ^b^	−10.33 ± 1.72 ^b^	86.4 ± 0.51 ^a^
2.0% IN-6.0% PPI-ACN-NLs	270.2 ± 0.66 ^d^	0.32 ± 0.003 ^c^	−15.76 ± 0.81 ^d^	88.6 ± 0.71 ^a^
4.0% IN-6.0% PPI-ACN-NLs	280.8 ± 0.89 ^c^	0.37 ± 0.015 ^b^	−18.25 ± 0.71 ^e^	84.1 ± 0.95 ^b^
6.0% IN-6.0% PPI-ACN-NLs	295.0 ± 2.37 ^b^	0.42 ± 0.020 ^a^	−15.20 ± 1.33 ^d^	83.7 ± 0.27 ^c^
8.0% IN-6.0% PPI-ACN-NLs	322.3 ± 1.07 ^a^	0.41 ± 0.008 ^a^	−12.48 ± 0.18 ^b^	82.5 ± 0.23 ^c^
10.0% IN-6.0% PPI-ACN-NLs	337.2 ± 3.51 ^a^	0.43 ± 0.030 ^a^	−14.90 ± 0.97 ^c^	80.0 ± 0.87 ^d^

**Table 3:** Fitting results of three nanoliposomes in different slow-release kinetic models during simulated gastric digestion.

Sample	Mathematical Model	Fitting Formula	R^2^
ACN-NLs	$\frac{M_{t}}{M_{0}}=kt$	$\frac{M_{t}}{M_{0}}=0.23t+9.97$	0.8764
	$\ln\left(1-\frac{M_{t}}{M_{0}}\right)=-kt$	$\frac{M_{t}}{M_{0}}=32.02(1-e^{0.03t})$	0.9873
	$\frac{M_{t}}{M_{0}}=kt^{0.5}$	$\frac{M_{t}}{M_{0}}=2.84t^{0.5}+1.96$	0.9213
	$\frac{M_{t}}{M_{0}}=kt^{n}$	$\frac{M_{t}}{M_{0}}=4.20t^{0.41}$	0.9080
PPI-ACN-NLs	$\frac{M_{t}}{M_{0}}=kt$	$\frac{M_{t}}{M_{0}}=0.07t+5.60$	0.8931
	$\ln\left(1-\frac{M_{t}}{M_{0}}\right)=-kt$	$\frac{M_{t}}{M_{0}}=13.26(1-e^{0.04t})$	0.9448
	$\frac{M_{t}}{M_{0}}=kt^{0.5}$	$\frac{M_{t}}{M_{0}}=1.14t^{0.5}+2.23$	0.9858
	$\frac{M_{t}}{M_{0}}=kt^{n}$	$\frac{M_{t}}{M_{0}}=1.94t^{0.41}$	0.9282
IN-PPI-ACN-NLs	$\frac{M_{t}}{M_{0}}=kt$	$\frac{M_{t}}{M_{0}}=0.08t+7.12$	0.8574
	$\ln\left(1-\frac{M_{t}}{M_{0}}\right)=-kt$	$\frac{M_{t}}{M_{0}}=14.71(1-e^{0.40t})$	0.8935
	$\frac{M_{t}}{M_{0}}=kt^{0.5}$	$\frac{M_{t}}{M_{0}}=1.14t^{0.5}+3.55$	0.9339
	$\frac{M_{t}}{M_{0}}=kt^{n}$	$\frac{M_{t}}{M_{0}}=2.95t^{0.35}$	0.9164

Note: M_t_ and M_0_ represent the actual and theoretical release amounts of ACN at time t, k is the constant coefficient of the equation, n is the release index used to characterize the release mechanism, and R is the correlation coefficient. Anthocyanin nanoliposomes (ACN-NLs); pea protein isolate-coated ACN-NLs (PPI-ACN-NLs); inulin-coated pea protein isolate–ACN-NLs (IN-PPI-ACN-NLs).

**Table 4:** Fitting results of three nanoliposomes in different slow-release kinetic models during simulated intestinal digestion.

Sample	Mathematical Model	Fitting Formula	R^2^
ACN-NLs	$\frac{M_{t}}{M_{0}}=kt$	$\frac{M_{t}}{M_{0}}=0.17t+15.76$	0.8983
	$\ln\left(1-\frac{M_{t}}{M_{0}}\right)=-kt$	$\frac{M_{t}}{M_{0}}=28.90(1-e^{0.06t})$	0.9095
	$\frac{M_{t}}{M_{0}}=kt^{0.5}$	$\frac{M_{t}}{M_{0}}=1.93t^{0.5}+10.72$	0.9399
	$\frac{M_{t}}{M_{0}}=kt^{n}$	$\frac{M_{t}}{M_{0}}=10.75t^{0.23}$	0.9718
PPI-ACN-NLs	$\frac{M_{t}}{M_{0}}=kt$	$\frac{M_{t}}{M_{0}}=0.22t+10.98$	0.9169
	$\ln\left(1-\frac{M_{t}}{M_{0}}\right)=-kt$	$\frac{M_{t}}{M_{0}}=32.13(1-e^{0.03t})$	0.9803
	$\frac{M_{t}}{M_{0}}=kt^{0.5}$	$\frac{M_{t}}{M_{0}}=2.86t^{0.5}+1.94$	0.9870
	$\frac{M_{t}}{M_{0}}=kt^{n}$	$\frac{M_{t}}{M_{0}}=4.1t^{0.43}$	0.9826
IN-PPI-ACN-NLs	$\frac{M_{t}}{M_{0}}=kt$	$\frac{M_{t}}{M_{0}}=0.14t+12.94$	0.8335
	$\ln\left(1-\frac{M_{t}}{M_{0}}\right)=-kt$	$\frac{M_{t}}{M_{0}}=24.47(1-e^{0.05t})$	0.9021
	$\frac{M_{t}}{M_{0}}=kt^{0.5}$	$\frac{M_{t}}{M_{0}}=1.80t^{0.5}+7.04$	0.9340
	$\frac{M_{t}}{M_{0}}=kt^{n}$	$\frac{M_{t}}{M_{0}}=6.76t^{0.28}$	0.9158

Note: M_t_ and M_0_ represent the actual and theoretical release amounts of ACN at time t, k is the constant coefficient of the equation, n is the release index used to characterize the release mechanism, and R is the correlation coefficient. Anthocyanin nanoliposomes (ACN-NLs); pea protein isolate-coated ACN-NLs (PPI-ACN-NLs); inulin-coated pea protein isolate–ACN-NLs (IN-PPI-ACN-NLs).

## Data Availability

The original contributions presented in the study are included in the article, further inquiries can be directed to the corresponding author.
